# A Case of a Previously Unreported Drainage of the Maxillary Vein

**DOI:** 10.7759/cureus.35272

**Published:** 2023-02-21

**Authors:** Andrew B. Wang, Joe Iwanaga, Juan J Cardona, Łukasz Olewnik, Samir Anadkat, R. Shane Tubbs

**Affiliations:** 1 Department of Medicine, Tulane University School of Medicine, New Orleans, USA; 2 Department of Neurosurgery, Tulane University School of Medicine, New Orleans, USA; 3 Department of Anatomy, Kurume University School of Medicine, Kurume, JPN; 4 Department of Dental and Oral Surgery, Kurume University School of Medicine, Kurume, JPN; 5 Department of Neurology, Tulane University School of Medicine, New Orleans, USA; 6 Department of Anatomical Dissection and Donation, Medical University of Lodz, Lodz, POL; 7 Department of Structural & Cellular Biology, Tulane University School of Medicine, New Orleans, USA; 8 Department of Anatomical Sciences, St. George’s University, St. George’s, GRD; 9 Department of Surgery, Tulane University School of Medicine, New Orleans, USA; 10 Department of Neurosurgery, Ochsner Neuroscience Institute, New Orleans, USA

**Keywords:** tributary, maxillary vein, facial vein, cadaver, anatomy

## Abstract

Due to the importance of venous drainage of the head and neck in various pathological conditions, knowledge of anatomical variations is important to the clinician. Here we report a case of an unusual drainage pattern of the maxillary vein. A tributary of the left maxillary vein was found in a female cadaver (72 years old at the time of death) to travel through the medial aspect of the ramus of the mandible via an accessory mandibular foramen, which drains into the ipsilateral facial vein slightly proximal to the point where the anterior branch of the retromandibular and facial veins merged to form the left common facial vein. The diameter of the variant vein at the junction with the maxillary vein and at the junction with the facial vein was 1.0 mm and 1.1 mm, respectively. We report a previously unreported variant of the maxillary vein bypassing the retromandibular vein and draining directly into the facial vein. Knowledge of such a variant might help explain various complications such as hemorrhage and might prevent iatrogenic injury of the blood vessels during surgery in this region.

## Introduction

The maxillary vein drains part of the blood from the pterygoid venous plexus to the retromandibular vein. The pterygoid venous plexus drains the nasopharynx, nasal cavity, and paranasal sinuses, and drains from the infraorbital vein anteriorly and the inferior alveolar vein inferiorly. The maxillary vein then joins with the superficial temporal vein to form the retromandibular vein [1,2]. The retromandibular vein combines with the common facial vein to drain into the internal jugular vein [1,2]. Given the importance of the confluence into the internal jugular vein, the maxillary vein is vital for the venous drainage of the head and neck.

The maxillary vein accompanies the maxillary artery [3]. It runs from behind the neck of the mandible and is derived from multiple tributaries of the pterygoid plexus. Having variable presentations, the maxillary artery and vein may run inside or outside superficial or deep to the lateral pterygoid muscle [4]. Formed at the confluence of the superficial temporal and maxillary veins, the retromandibular vein lies medial to the facial nerve [5]. There are various morphologies regarding the relationship between the retromandibular vein and the mandible ramus, which could influence the intraoperative bleeding risk during sagittal split ramus ostomies [6]. Since the facial vein and the retromandibular vein serve as important landmarks in magnetic resonance imaging (MRI) and computed tomography (CT) imaging, the maxillary vein’s proximity to the mandibular ramus and its same level at the lingula of the mandible are important for oral surgeries such as repairing mandibular deformities [3]. Surgeons should be aware of such venous anatomy when conducting orthognathic surgeries in order to prevent unnecessary hemorrhage when resecting the coronoid process [3].

While it is assumed that the maxillary vein drains into the retromandibular vein, here, we describe a variant drainage of the maxillary vein into the ipsilateral facial vein.

## Case presentation

**Figure 1:**
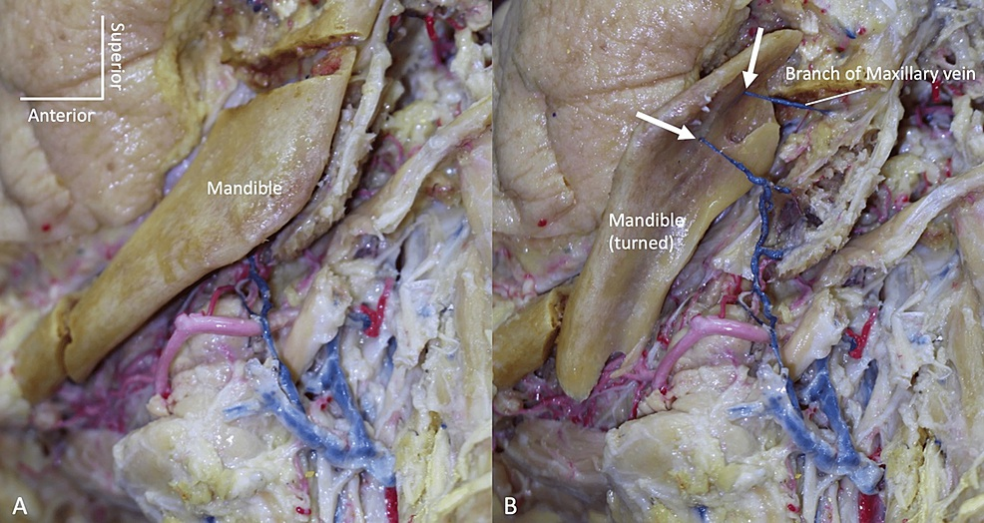
A small tributary from the left maxillary vein is seen traveling through the medial aspect of the ramus of the mandible. Note that the variant vein enters and exits from the mandible via accessory mandibular foramina (arrows). (A) The mandible is cut but still in place. (B) The mandible is turned laterally to illustrate the variant vein.

**Figure 2:**
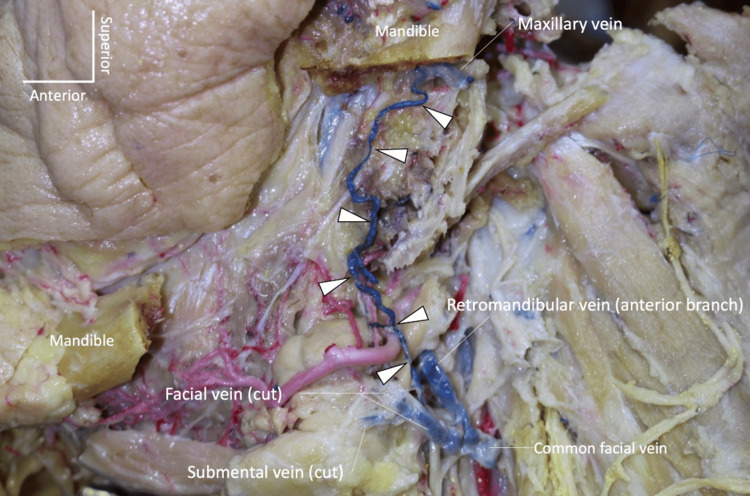
The variant vein (arrowheads) drains into the facial vein. Note that most parts of the ramus of the mandible and posterior part of the body of the mandible have been removed.

During the routine dissection of the head and neck, a variant vein was found in an adult formalin-fixed human cadaver whose age at death was 72 years. A small tributary from the left maxillary vein traveled through the medial aspect of the ramus of the mandible (via an accessory mandibular foramen, (Figure 1) and drained into the ipsilateral facial vein slightly above the point where the anterior branch of the retromandibular and facial veins merged to form the left common facial vein (Figure 2).

The diameter of the variant vein at the junction with the maxillary vein and at the junction with the facial vein was 1.0 mm and 1.1 mm, respectively. The inferior alveolar vein was in its normal position as was the common facial vein. No obvious surgical scar or anatomical variation was found in the dissected area.

The authors state that every effort was made to follow all local and international ethical guidelines and laws that pertain to the use of human cadaveric donors in anatomical research [7].

## Discussion

We report a case of a variant branch of the maxillary vein that drained into the ipsilateral facial vein after coursing through accessory foramina (Figure 1). To our knowledge, such an anatomical venous variation has not been previously reported.

The embryology of the head and neck veins arises on Carnegie stage 10 (day 27), with the ventral pharyngeal vein arising from the anterior cardinal vein. As the neck elongates, the pharyngeal drains into the future internal jugular vein through the developing mandibular and hyoid pharyngeal arches. On Carnegie stage 16 (day 39), the ventral pharyngeal moves to drain into the internal jugular vein. The primitive maxillary vein develops cranial and lateral to the ventral pharyngeal vein. The primitive maxillary vein develops to drain the orbital area, but as it elongates it will eventually drain the areas supplied by the trigeminal nerve. By Carnegie stage 20 (day 49), the primitive maxillary vein extends and anastomoses with the linguofacial vein. On Carnegie stage 20 (days 53 to 58), the primitive maxillary vein will become the common facial vein. It will also receive blood from the retromandibular vein. A plexiform anastomosis between lateral tributaries of the linguofacial and maxillary veins then forms around the outer labial margin. In addition, among the many plexiform and more medial tributaries of the maxillary vein, the adult pterygoid plexus is formed [8-10].

Previously, a variant of the maxillary vein was described, where in the absence of the retromandibular vein, a maxillary vein split anteriorly and posteriorly connecting directly to an atypical external jugular vein and facial vein, respectively [11,12]. Another tributary that empties into the facial vein is the external palatine vein, which is well known as “paratonsillar vein” for ENT surgeons but has not been well studied [13]. This drains the venous blood from the palatine tonsil.

Common surgical applications include the bilateral split osteotomy, the most performed jaw surgery where the mandible is split bilaterally to restore proper dentofacial function [6]. This process requires incision and dissection into the intraoral area, especially in the pterygomandibular space. Injury to the inferior alveolar neurovascular bundles, the maxillary artery, and pterygoid venous plexus should be avoided [8,14,15].

Similar head and neck neurovasculature variants have also been reported in the formation of the retromolar canal and foramen. The retromolar fossa is a variating foramen on the ramus of the mandible behind the third molar that drains into the retromolar canal [16]. In a case report of a cadaveric specimen with a bifid mandibular canal, the superior canal contained the inferior alveolar nerve and artery, and the inferior canal contained a large inferior alveolar vein [17].

These variations can be observed on cone-beam CT (CBCT) images but can be missed [18,19]. It is important to further examine the anatomy of the maxillary vein in order to standardize anatomical variants and provide archival information so that future encounters with such a variant can be better evaluated.

Declaration

The authors sincerely thank those who donated their bodies to science so that anatomical research could be performed. Results from such research can potentially increase mankind’s overall knowledge, which can then improve patient care. Therefore, these donors and their families deserve our highest gratitude [20].

## Conclusions

We report a previously unreported variant of the maxillary vein bypassing the retromandibular vein and draining directly into the facial vein. Knowledge of such a variant might help explain various complications and might prevent iatrogenic complications during surgery in this region.

## References

[1] Golub B, Bordoni B (2022). Neuroanatomy, pterygoid plexus. StatPearls [Internet].

[2] von Arx T, Tamura K, Yukiya O, Lozanoff S (2018). The face - a vascular perspective. A literature review. Swiss Dent J.

[3] Odaka K, Matsunaga S (2020). Course of the maxillary vein and its positional relationship with the mandibular ramus require attention during mandibuloplasty. J Craniofac Surg.

[4] Bassiri Gharb B, Frautschi RS, Halasa BC (2017). Watershed areas in face transplantation. Plast Reconstr Surg.

[5] El Kininy W, Davy S, Stassen L, Barry DS (2018). Novel variations in spatial relations between the facial nerve and superficial temporal and maxillary veins. Folia Morphol (Warsz).

[6] Sugahara K, Matsunaga S, Yamamoto M (2020). Retromandibular vein position and course patterns in relation to mandible: anatomical morphologies requiring particular vigilance during sagittal split ramus osteotomy. Anat Cell Biol.

[7] Iwanaga J, Singh V, Takeda S (2022). Standardized statement for the ethical use of human cadaveric tissues in anatomy research papers: Recommendations from Anatomical Journal Editors-in-Chief. Clin Anat.

[8] Iwanaga J, Ibaragi S, Okui T, Hur MS, Kageyama I, Tubbs RS (2022). An anatomical study of the blood supply to the mylohyoid muscle: the so-called "mylohyoid branch" of the inferior alveolar artery is an arterial anastomosis. Ann Anat.

[9] Ono K, Yoshioka N, Hage D, Ibaragi S, Tubbs RS, Iwanaga J (2021). Duplication of the external jugular vein: a language barrier of database search in classic anatomical studies. Surg Radiol Anat.

[10] Som PM, Berenstein A (2018). The development of the arteries and veins of the head and neck. Neurographics.

[11] Patil J, Kumar N, Swamy RS, D'Souza MR, Guru A, Nayak SB (2014). Absence of retromandibular vein associated with atypical formation of external jugular vein in the parotid region. Anat Cell Biol.

[12] Manta MD, Jianu AM, Rusu MC, Popescu ŞA (2021). Launay's external carotid vein. Medicina (Kaunas).

[13] Téllez-Hernández LV, Tibaduiza-Rodriguez IA, Ferreira-Arquez H (2019). Unilateral anatomical variation in the venous drainage of face and neck. Int J Pharmaceut Res.

[14] Iwanaga J, Kikuta S, Ibaragi S, Watanabe K, Kusukawa J, Tubbs RS (2020). Clinical anatomy of the accessory mandibular foramen: application to mandibular ramus osteotomy. Surg Radiol Anat.

[15] Monson LA (2013). Bilateral sagittal split osteotomy. Semin Plast Surg.

[16] Kikuta S, Iwanaga J, Nakamura K, Hino K, Nakamura M, Kusukawa J (2018). The retromolar canals and foramina: radiographic observation and application to oral surgery. Surg Radiol Anat.

[17] Iwanaga J, Wilson C, Simonds E (2018). First report of a bifid mandibular canal containing a large vein draining into the anterior jugular vein. Kurume Med J.

[18] Iwanaga J, Takeshita Y, Matsushita Y, Hur MS, Ibaragi S, Tubbs RS (2022). What are the retromolar and bifid/trifid mandibular canals as seen on cone-beam computed tomography? Revisiting classic gross anatomy of the inferior alveolar nerve and correcting terminology. Surg Radiol Anat.

[19] Kumar Potu B, Jagadeesan S, Bhat KM, Rao Sirasanagandla S (2013). Retromolar foramen and canal: a comprehensive review on its anatomy and clinical applications. Morphologie.

[20] Iwanaga J, Singh V, Ohtsuka A (2021). Acknowledging the use of human cadaveric tissues in research papers: recommendations from anatomical journal editors. Clin Anat.

